# Randomized Phase II Trial to Compare the Efficacy of Haloperidol and Olanzapine in the Control of Chemotherapy-Induced Nausea and Vomiting in Nepal

**DOI:** 10.1200/JGO.18.00245

**Published:** 2019-04-23

**Authors:** Soniya Dulal, Bishnu Dutta Paudel, Prakash Neupane, Aarati Shah, Bibek Acharya, Bishesh Sharma Poudyal, Ramila Shilpakar, Lori Anne Wood

**Affiliations:** ^1^ National Academy of Medical Sciences, Bir Hospital, Kathmandu, Nepal; ^2^ The University of Kansas Medical Center, Kansas City, KS; ^3^ Civil Service Hospital, Kathmandu, Nepal; ^4^ Queen Elizabeth II Health Sciences Centre, Halifax, Nova Scotia, Canada

## Abstract

**PURPOSE:**

The purpose of the study was to compare efficacy and toxicity of olanzapine (OLN; a higher-cost drug) and haloperidol (HAL; a lower-cost drug) in the prevention of chemotherapy-induced nausea and vomiting (CINV) in patients who receive highly emetogenic chemotherapy (HEC).

**PATIENTS AND METHODS:**

In a randomized, phase II trial, patients were randomly assigned to receive either OLN 10 mg orally on days 1 to 4 or HAL 1 mg orally on day 1 and 0.5 mg twice daily on days 2 to 4. Both groups received ondansetron 16 mg and dexamethasone 12 mg intravenously on day 1. Patients recorded their nausea using the Edmonton Symptom Assessment Scale (ESAS) and recorded daily episodes of vomiting from day 1 to day 5. The primary end point was complete nausea prevention (CNP; ie, ESAS of 0). Secondary end point was complete emesis prevention (CEP).

**RESULTS:**

Sixty-five patients were randomly assigned, and 64 received their allocated treatment (n = 32 in each arm). There was no difference in CNP during the overall period (days 1 to 5) between OLN and HAL (68.7% *v* 71.8%; *P* = .78). In the acute period (day 1) and the delayed period (days 2 to 5), CNP was similar between OLN and HAL (acute: 84.3% *v* 81.2%; delayed: 68.7% *v* 75%). No difference was identified in the rate of CEP during the overall period (81.2% with OLN *v* 78.1% with HAL; *P* = .75), during the acute period (93.7% with OLN *v* 90.6% with HAL), or during the delayed period (84.3% with OLN *v* 84.3% with HAL). No difference in toxicities was noted between treatment arms.

**CONCLUSION:**

In this study, HAL had comparable efficacy to OLN in the management of CINV, which suggests that it is the higher-value option in patients who receive HEC in resource-scarce countries.

## INTRODUCTION

Chemotherapy-induced nausea and vomiting (CINV) has a great impact on the quality of life of patients who receive some cancer therapy.^1^ These adverse effects can also result in anorexia, decreased performance status, metabolic imbalance, wound dehiscence, and nutritional deficiency.^2,3^

CINV is commonly classified as acute, delayed, breakthrough, anticipatory, or refractory. Acute onset occurs within a few minutes to several hours after drug administration and commonly resolves in the first 24 hours, whereas delayed onset occurs more than 24 hours after chemotherapy administration.^4,5^ Breakthrough symptoms refers to nausea or vomiting that occurs despite prophylactic treatment.^6^ Chemotherapeutic agents are classified as having high, moderate, low, or minimal emetogenic potential depending on their risk of acute emesis with no prophylaxis.^7^

The principal neuroreceptors involved in the emetic response are the serotonin and dopamine receptors.^8,9^ Other neuroreceptors involved in emesis include acetylcholine, corticosteroid, histamine, cannabinoid, opiate, and neurokinin-1 (NK-1) receptors.^10^ Antiemetic agents can block different neuronal pathways or behave synergistically with other antiemetic agents to potentiate an antiemetic effect. Although vomiting can often be prevented by using prophylactic antiemetic regimens, nausea is harder to control.^11^ Pharmacologic treatment options for CINV exploit these receptors and include 5-HT_3_ receptor antagonists, NK-1 receptor antagonists, corticosteroids, dopamine receptor antagonist, benzodiazepines, atypical antipsychotics, and cannabinoids.

Olanzapine (OLN), an atypical antipsychotic, blocks multiple neurotransmitter receptors involved in CINV. It is effective at prevention of acute and delayed emesis caused by both highly emetogenic chemotherapy (HEC) and moderately emetogenic chemotherapy.^12^ OLN is now a recommended prophylactic drug in the updated ASCO guidelines for HEC.^13^ Haloperidol (HAL) is a butyrophenone with a high affinity for dopamine D2 receptors.^14^ It acts on the chemoreceptor trigger zone.

Studies have compared OLN-based antiemetics with other antiemetics, like NK-1 receptor antagonists. However, none have compared OLN with a less expensive medication, like HAL, as an antiemetic to control CINV. If HAL is as effective as OLN, it could have an important clinical and financial impact on patients from countries with limited resources without necessarily added toxicity and compromised efficacy. This would minimize the economic burden, particularly in the context of resource-poor countries, where cost and out-of-pocket expenses for patients are major concerns and limitations to oncology care.

To our knowledge, this is the first study to compare OLN and HAL for the prevention of CINV. The primary objective of this study was to compare the efficacy of OLN and HAL in the prevention of nausea caused by HEC, and the secondary objectives were to compare the efficacy of emesis prevention and to evaluate the safety and cost difference of OLN compared with HAL.

## PATIENTS AND METHODS

This was a randomized, phase II study conducted at the Department of Clinical Oncology, National Academy of Medical Sciences, Bir Hospital, Kathmandu, Nepal. Approval was obtained from the institutional review board of the National Academy of Medical Sciences, Bir Hospital, and informed written consent was obtained from each participant.

The study included adult patients age 18 years or older who were receiving HEC, had Eastern Cooperative Oncology Group performance status of 0 to 2, and who gave informed written consent. Exclusion criteria were patients with known hypersensitivity to OLN or HAL, with documented Parkinson disease, who had experienced extrapyramidal syndromes or intolerance in the past to olanzapine, who had notable comorbidities, and who had higher than grade 1 renal or liver dysfunction.^15^ Patients were chemotherapy naive, receiving HEC, defined as cisplatin doses of 70 mg/m^2^ or greater or as cyclophosphamide 500 mg/m^2^ or greater and doxorubicin 50 mg/m^2^ or greater.

Random assignment was 1:1 using a simple random assignment method. Sixty-five envelopes were marked as either arm A or arm B, and patients blindly chose one envelope.

### Study Treatment and Assessment

Patients in arm A (OLN) received 10 mg of oral OLN 30 to 60 minutes before chemotherapy on day 1 and then daily from day 2 to day 4. Patients in arm B (HAL) received 1 mg of oral HAL 30 to 60 minutes before chemotherapy on day 1 and then 0.5 mg twice a day from day 2 to day 4. All patients received ondansetron 16 mg and dexamethasone 12 mg intravenously 30 to 60 minutes before chemotherapy on day 1. Use of oral lorazepam 0.5 to 2 mg every 6 hours as needed was allowed as a breakthrough antiemetic for CINV refractory to the assigned treatment arm.^16^ The protocol was continued with each chemotherapy cycle for six cycles.

Patients recorded their nausea using the Edmonton Symptom Assessment Scale (ESAS) from days 1 to 5. They recorded their daily episodes of vomiting (number and timing) and the use of additional antiemetics. They also recorded any adverse effects. Toxicities were graded using the Common Terminology Criteria for Adverse Events, version 4.0.^15^ For safety monitoring, blood sugar levels, a lipid profile, liver function tests, and an ECG were done before and after each chemotherapy cycle.

### Study End Points

The primary end point was complete nausea prevention, measured as 0 on the ESAS. The secondary end point was complete emesis prevention without the use of additional antiemetics. These end points were analyzed in the acute period (day 1), delayed period (days 2 to 5), and overall period (acute and delayed, or days 1 to 5).

### Data Collection and Statistical Analysis

Data collection was done on a standardized data collection sheet. Patient demographics and clinical characteristics, such as age, sex, and Eastern Cooperative Oncology Group performance status, were obtained along with date of enrollment, chemotherapy cycle, treatment regimen. Symptom assessment was recorded using ESAS to measure the presence and intensity of symptoms.

The sample size was calculated on the basis of a confidence level of 95%, a confidence interval of 10%, and an expected complete nausea control rate of 84%. The calculated sample size was 52; with an assumed drop-out rate of 25%, the plan was to enroll 65 patients. Patients who had any ESAS or diary information completed were included in the analysis.

Frequencies and percentages were obtained for each categoric variable. The mean and median were obtained for continuous variables. Comparisons between the two groups were assessed using the χ^2^ test. Subgroup analysis was performed using crosstabs to evaluate the study outcomes. A two-tailed level of significance at a *P* value of less than .05 was considered significant and was applied to all statistical tests. SPSS, version 24, statistical software (SPSS, Chicago, IL) was used for statistical analyses.

## RESULTS

**FIG 1:**
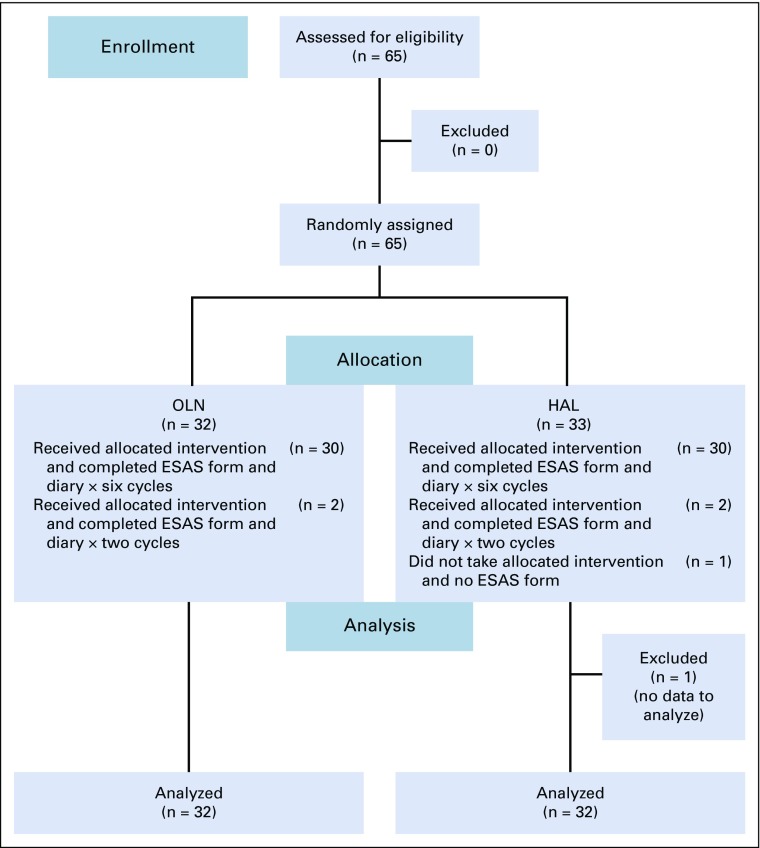
CONSORT: Distribution and random assignment of study patients. ESAS, Edmonton Symptom Assessment Scale; HAL, haloperidol; OLN, olanzapine.

A total of 65 patients were randomly assigned from April 1, 2017, to December 25, 2017. Complete data for all six cycles of chemotherapy were obtained for 60 patients, data for the first two cycles only were obtained for four patients (n = 2 in each arm), and no data were obtained for one patient who did not receive the allocated treatment in the HAL arm. Therefore, 64 patients were analyzed, 32 in each arm (Fig 1).

Patient demographics of the study population are listed in Table 1. There were no statistically significant differences between the two groups. The median age of the study population was 53 years, and women represented 51.5% of the population. Lung cancer accounted for 43.7% of the cancer diagnoses; breast and hematologic malignancies were the next most common.

**TABLE 1:**
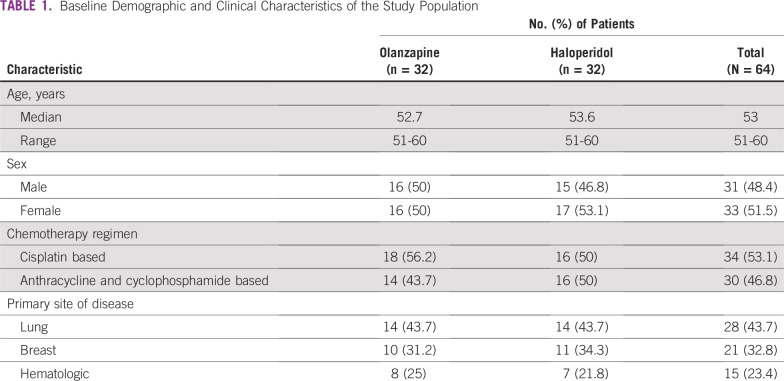
Baseline Demographic and Clinical Characteristics of the Study Population

**FIG 2:**
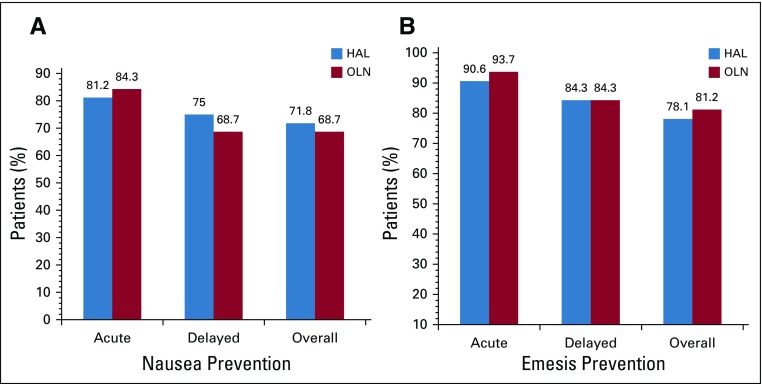
Percentage of patients with (A) complete nausea prevention and (B) complete emesis prevention. HAL, haloperidol; OLN, olanzapine.

With regard to the primary end point of complete nausea prevention, there was no difference between OLN and HAL during the overall period (68.7% *v* 71.8%; *P* = .78; Table 2; Fig 2A). In both the acute period and the delayed period, complete nausea prevention was similar between OLN and HAL (acute: 84.3% *v* 81.2%; delayed: 68.7% *v* 75%). In the OLN arm, nausea was experienced by no patients in the acute period, by five patients in the delayed period, and by five patients in both the acute and delayed period for a total of 10 patients (31.3%). In the HAL arm, nausea was experienced by one patient in the acute period, by three patients in the delayed period, and by five patients in both the acute and delayed period for a total of nine patients (28.1%).

**TABLE 2:**
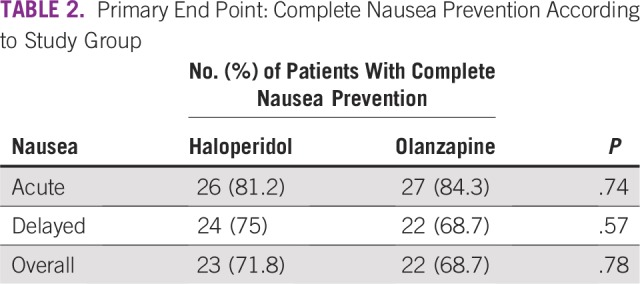
Primary End Point: Complete Nausea Prevention According to Study Group

With regard to the secondary end point of complete emesis prevention, there was no difference between OLN and HAL during the overall period (81.2% OLN *v* 78.1% HAL; *P* = .75) nor in the acute period (93.7% OLN *v* 90.6% HAL) or the delayed period (84.3% OLN *v* 84.3% HAL; Table 3; Fig 2B). In the OLN arm, emesis was experienced by one patient in the acute period, by four patients in the delayed period, and by one patient in both the acute and delayed period for a total of six patients (18.8%). In the HAL arm, emesis was experienced by two patients in the acute period, by four patients in the delayed period, and by one patient in both the acute and delayed period for a total of seven patients (21.9%).

**TABLE 3:**
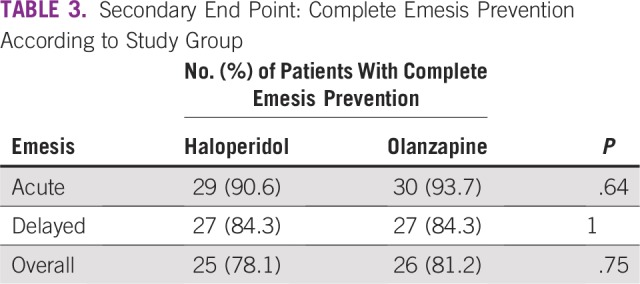
Secondary End Point: Complete Emesis Prevention According to Study Group

There was no difference in the number of patients who required lorazepam for breakthrough nausea between the two arms nor in the amount of lorazepam used. The most common adverse events in the HAL arm were grade 1 drowsiness (9.3%) and dry mouth (6.2%). In the OLN arm, grade 1 headache, constipation, hyperglycemia, and dry mouth were each seen in 6.2% of patients, and drowsiness was noted in 3.1% patients.

From a cost point of view, a tablet of HAL 1 mg in Nepal is less expensive than OLN 10 mg (rupees: 2 *v* 18; personal communication with Bir Hospital Pharmacy, October 2018). The total cost of HAL versus OLN per cycle was 8 versus 72 rupees. Thus, the use of HAL led to a cost reduction of approximately 64 rupees per cycle, which equates to 384 rupees over six cycles.

## DISCUSSION

CINV continues to be a common clinical challenge in oncology, especially in developing countries where not all drugs are available and out-of-pocket drug costs can be prohibitory. Antiemetics used in the prevention of CINV are often expensive and add greatly to the overall cost of treatment. Studies have shown, however, that CINV can adversely affect quality of life, lead to change in treatment plans, and increase the use of health care resources.^17-19^

This study compared OLN with HAL and showed comparable control in both nausea and emesis during the overall, acute, and delayed periods. These encouraging results also showed that the majority of patients treated with HEC in a tertiary public hospital in Nepal experienced complete nausea and emesis control with either arm of the study. These nausea and emesis control rates are comparable to those of recent studies done in other countries.^20,21^

Despite our results, HAL is not recommended as an antiemetic in the ASCO guidelines.^22^ It is recommended only as a treatment option for breakthrough CINV in the National Comprehensive Cancer Network guidelines.^23^

The fact that HAL, a less expensive drug, appears to have similar nausea and emesis control as OLN is practice changing. HAL is also a more accessible drug in developing countries, especially in rural areas. In this study, the cost savings to an individual patient for six cycles of chemotherapy was 384 rupees. Although one may point out this is only 3.45 US dollars, it is important to remember that 25% of the Nepalese population lives below the poverty line; thus any cost savings has an impact.^24^ In Nepal, a large proportion of patients diagnosed with cancer belong to this low socioeconomic group, often are from rural areas, and often delay or forego treatment because of the high costs involved.

We acknowledge that a limitation of this study was its small size. However, given these initial positive results and the possibility of notable cost savings, additional larger, randomized trials in developed countries seem warranted, because value-based care is important in all countries. In fact, the cost savings may be far greater in developed countries, where the cost difference between these two drugs is even more substantial. HAL 1 mg costs 0.45 US dollars, and OLN 10 mg costs 24.32 US dollars.^25^ Per cycle, this would be 1.80 US dollars for HAL versus 97.28 US dollars for OLN—a difference of 572.88 US dollars for six cycles.

According to the results of this study, the use of HAL to prevent CINV in this patient population is safe, effective, and less expensive than OLN. Its use as standard of care is warranted in Nepal and also may be warranted in developed countries.
